# Changes in obesity-related diseases and biochemical variables after laparoscopic sleeve gastrectomy: a two-year follow-up study

**DOI:** 10.1186/1471-2482-14-8

**Published:** 2014-02-11

**Authors:** Villy Våge, Vetle Aaberge Sande, Gunnar Mellgren, Camilla Laukeland, Jan Behme, John Roger Andersen

**Affiliations:** 1 Department of Surgery, Førde Central Hospital, 6807 Førde, Norway; 2 Department of Clinical Science, University of Bergen, 5020 Bergen, Norway; 3 Department of Health, Sogn og Fjordane University College, 6803 Førde, Norway; 4 Hormone Laboratory, Haukeland University Hospital, 5021 Bergen, Norway

**Keywords:** Sleeve gastrectomy, Obesity, Comorbidities, Complications

## Abstract

**Background:**

To evaluate changes in obesity-related diseases and micronutrients after laparoscopic sleeve gastrectomy (LSG).

**Methods:**

We started the procedure in May 2007, and by December 2011, 117 patients could be evaluated for a two year follow-up. Comparisons of preoperative status with 12 and 24 months postoperative status were made for body mass index (BMI), obesity-related diseases and micronutrients.

**Results:**

Major complications included bleeding requiring transfusion at 5.1%, leak at 1.7% and abscess without a visible leak at 0.9%. Mean BMI was reduced from 46.6 (standard deviation (SD) 6.0) kg/m^2^ to 30.6 (SD 5.6) kg/m^2^ at two years, and resolution occurred for 80.7% of patients with type 2 diabetes, 63.9% with hypertension, 75.8% with hyperlipidemia, 93.0% with sleep apnea, 31.4% with musculoskeletal pain, 85.4% with snoring and 73.3% with urinary incontinence. Amenorrhea resolved in all premenopausal females. The proportion of patients with symptomatic gastroesophageal reflux disease increased from 12.8% to 27.4%. The prevalence of patients with low ferritin-levels increased, while 25-hydroxyvitamin D (25(OH)D) deficiency decreased postoperatively.

**Conclusions:**

LSG is an effective procedure for morbid obesity and obesity-related diseases, but the technique should be further explored particularly to avoid gastroesophageal reflux.

## Background

Between 1980 and 2008, the age-standardized mean global body mass index (BMI) increased by 0.4–0.5 kg/m^2^ per decade in men and women [1], and worldwide obesity more than doubled. Obesity, and particularly morbid obesity (BMI ≥ 40) is known as a strong risk factor for several diseases and premature death [2].

Bariatric surgery is the only evidence-based treatment of morbid obesity with proven, sustained weight loss and improvement in comorbidities [3-5]. Laparoscopic sleeve gastrectomy (LSG) was introduced as the first stage in a two-staged bariatric surgical approach on super-obese or high-risk patients [6], but has now gained acceptance as a stand-alone bariatric procedure [7-11]. Physiologically it is an attractive procedure because it reduces the gastric volume while preserving the continuity of the gastrointestinal tract. Data on complications and weight loss after LSG have been increasingly published in the surgical literature, but data for the effects on comorbidities and micronutrients should be further explored [12,13].

Our bariatric surgical program started in 2001 with open Biliopancreatic diversion with duodenal switch (BPDDS), and LSG as a stand-alone procedure was started in May 2007. We had no experience with laparoscopic bariatric surgery prior to May 2007, and this prospective study reviews our first patients focusing on procedure complications, comorbidity resolution and changes in biochemical variables at 12 and 24 months postoperatively.

## Methods

After having obtained written informed consent from the patients, data was prospectively collected and stored in our database from the first LSG in May 2007 when LSG was introduced as a part of our standard bariatric program. The database is part of our continuous surveillance-program and approved by the Norwegian Data Inspectorate. This present study is a prospective cohort study with data extracted from the database. By December 2011 we had 117 patients eligible for a two year follow-up. Indications for LSG were either a BMI ≥ 40 kg/m^2^ or a BMI ≥ 35 kg/m^2^ with obesity-related diseases. Contraindications for operation were alcohol or drug abuse and active psychosis.

Preoperative evaluation and care included a one day seminar with information about morbid obesity, bariatric surgery and its risks, and estimated results as well as projected possibilities about life changes after surgery. This was followed by an individual consultation with the bariatric surgeon and other health**-**personnel if needed. Preoperative advice included smoking cessation, increased physical activity and weight loss.

On the evening before the operation all patients received low molecular weight heparin subcutaneously (enoxaparin), 40 mg if < 160 kg or 60 mg if ≥ 160 kg, and an H_2_ blocker (cimetidine 300 mg) orally. Intravenous antibiotic prophylaxis (400 mg doxycycline, 1.5 g metronidazole) was started just prior to the operation. The operation was performed through six ports. Pneumoperitoneum was established through the upper part of the left rectal sheet using a 10 mm port containing the camera and the CO2-insufflator. A 15 mm port was introduced at the same level through the right rectal sheet. Four 5 mm ports were used: One at the right subcostal area, one just below the xiphoid process and two towards the left subcostal area. All ports were reusable (Karl Storz™) except for the 15 mm port which was non-reusable (Ethicon™).

The greater curvature was freed from the pylorus to the cardia, dividing all vessels by Ligasure (Covidien™). To ensure a good overview of the left crus and the gastro esophageal junction the periesophageal fat-pad was generally freed from both the diaphragm and the cardia. The stomach was divided along a 32 Fr bougie by the Tri-Stapler (Covidien™) from 1-2 cm proximal to the pylorus to the cardia. Over-sewing of the staple line was performed for visible bleeding. Attention was paid to avoid twisting or otherwise disrupting the gastric tube. The resected part of the stomach was removed without a bag through the incision for the 15 mm port. The abdominal fascia at this point was closed by two Polydioxanon (PDS) number 1 sutures. All skin-incisions were closed by intracutaneous reabsorbable sutures. Patients were allowed to drink freely from the first postoperative day, and discharged when tolerating a liquid diet. The enoxaparin was continued for ten days after discharge.

Postoperative advice included a low carbohydrate - high protein diet, intake of one multivitamin tablet daily, high frequency of water intake and physical activity. The first 61 patients were also routinely recommended to take Calcigran Forte (NycoMed Pharma™) containing one gram of calcium carbonate and 800IE 25-hydroxyvitamin D (25(OH)D) daily. Controls and data collection took place at the outpatient clinic 3, 12 and 24 months postoperatively. In addition**,** the patients were advised to see their general practitioner at 6 and 18 months. Pregnancy was strongly discouraged during the first 12 months after the operation.

Surgical complications were defined as complications occurring within 90 days after the surgical procedure. Obesity-related diseases were defined as diseases that were under medical care, and considered resolved when the patient no longer needed medical care for the actual disease (dichotomous variables). Diseases evaluated were type 2 diabetes mellitus (T2DM), hypertension, hyperlipidemia, sleep apnea, obstructive lung disease, musculoskeletal pain, anxiety, depression and gastroesophageal reflux. In addition, obesity-related problems as snoring, urinary leakage, amenorrhea and infertility were included independently of whether the patient received treatment or not. Infertility was defined as attempting to get pregnant over a period of two years without success.

Biochemical variables were selected according to our empirical experience, and were all analyzed by the Department of Clinical Biochemistry at our hospital except for the vitamin D-analyses (Hormone Laboratory, Haukeland University Hospital). The biochemical variables were converted into dichotomous variables, as either within the reference range or outside the reference range.

Statistical Package for the Social Sciences (SPSS) version 19.0 was used to perform the statistical analysis. Paired t- test was used in comparing paired means for change in BMI, and the McNemar´s test was used for categorical variables. Statistical significance was set conventionally at p < 0.05.

## Results

For the 117 patients studied (87 women and 30 men), the mean weight prior to the operation was 135.6 kg ± 23.7 kg (standard deviation (SD)), mean BMI 46.6 ± 6.0 kg/m^2^, and mean age 40.3 ± 10.7 years (Table 1).

**Table 1:** Overview of the patients (n = 117)

**Variables**	**Mean ± SD**
Age (years)	40.3 ± 10.7
Sex (Women/Men)	87/30
Weight (kg)	135.6 ± 23.7
BMI (kg/m^2^)	46.6 ± 6.0
Operation time (min)	134.6 ± 27.5
Concurrent operations*	4

SD = Standard deviation. BMI = body mass index.

*Concurrent operations were cho ***l*** *ecystectomi (2), appendectomy (1) and hiatal hernia repair (1).*

### Complications

Major complications included bleeding (5.1%, n = 6), leak (1.7%, n = 2) and abscess without a visible leak (0.9%, n = 1) (Table 2). One patient had both bleeding and leak. The two patients who had a leak were treated with nil by mouth and a nasojejunal feeding tube for two and five months respectively before it healed. Both leaks were at the cardiac region. Two of the patients with bleeding had relaparoscopy with evacuation of blood (1.7%). There was no conversion of laparoscopic to open surgery and no mortality.

**Table 2:** 90-day morbidity after surgery (n = 117)

**Complications**	**Patients, n (%)**
Bleeding (given transfusion)	6 (5.1%)
Reoperated	2 (1.7%)
Leak	2 (1.7%)
Reoperated	0 (0%)
Abscess without leak	1 (0.9%)
Reoperated	0 (0%)
*Minor complications:*	
Wound infection	3 (2.6%)
Incisional hernia	1 (0.9%)
Relaparoscopy for retained drain	1 (0.9%)

### Obesity-related diseases

LSG significantly lowered the BMI to 30.3 ± 5.9 kg/m2 and 30.6 ± 5.6 kg/m2 at 12 and 24 months respectively, and resolved obesity-related diseases (Table 3). At two years, the remission-rate for Type 2 Diabetes Mellitus (T2DM) was 80.7%, hypertension 63.9%, hyperlipidemia 75.8%, sleep apnoea 93.0%, musculoskeletal pain 31.4%, snoring 85.4% and urinary leakage 73.3%. Amenorrhea was resolved for all premenopausal female patients with two years data. We lacked two year data for three of our twelve patients with preoperative amenorrhea. The prevalence of gastroesophageal reflux disease (GERD) increased from 12.8% prior to the operation to 27.4% at two years (p = 0.011).

**Table 3:** Proportion of patients (%) with obesity-related diseases prior to, and at 12 and 24 months after laparoscopic sleeve gastrectomy

**Variable**	**Preoperative**	**12 months**	**24 months**	**P value**		
				12 vs 0 months	24 vs 0 months	24 vs 12 months
*No. with available data/total no.*	117/117	116/117	109/117			
BMI (kg/m^2^, mean ± SD)	46.6 ± 6.0	30.3 ± 5.9	30.6 ± 5.6	<0.001	<0.001	0.936
Treated for	*No. with disease/no. with available data (%)*					
T2DM	23/117 (19.7%)	5/114 (4.4%)	4/105 (3.8%)	<0.001	<0.001	1
Hypertension	50/117 (42.7%)	17/111 (15.3%)	16/104 (15.4%)	<0.001	<0.001	1
Hyperlipidemia	14/117 (12.0%)	5/114 (4.4%)	3/104 (2.9%)	0.039	0.021	1
Sleep apnea	15/117 (12.8%)	3/116 (2.9%)	1/109 (0.9%)	<0.001	<0.001	0.5
Obstructive lung disease	19/117 (16.2%)	19/114 (16.7%)	13/105 (12.4%)	1	0.063	0.063
Musculoskeletal pain	41/115 (35.7%)	29/114 (25.4%)	26/106 (24.5%)	0.067	0.026	0.804
Anxiety	18/117 (15.4%)	13/113 (11.5%)	11/103 (10.7%)	0.219	0.375	1
Depression	25/117 (21.4%)	18/113 (15.9%)	15/103 (14.6%)	0.109	0.125	1
GERD	15/117 (12.8%)	34/112 (30.4%)	29/106 (27.4%)	0.001	0.011	0.629
Suffering from (treated or not)						
Snoring	89/114 (78.1%)	28/112 (25%)	13/102 (12.8%)	<0.001	<0.001	0.002
*Premenopausal/total no. of women*	71/87	67/87	63/87			
Urinary leakage	33/87 (37.9%)	11/84 (13.1%)	8/79 (10.1%)	<0.001	<0.001	0.453
Amenorrhea	12/70 (17.1%)	3/65 (4.6%)	0/51 (0%)	0.021	0.016	1
Infertility	12/70 (17.1%)	*	4/52 (7.7%)		0.180	

BMI = Body mass index. SD = Standard deviation. No = Number. T2DM = Type 2 diabetes mellitus. GERD = Gastroesophageal reflux disease.

*Pregnancy was strongly discouraged during the first 12 months. At 24 months, two of the 12 women defined as infertile preoperatively had given birth to two healthy children.

### Biochemical variables

Of the biochemical variables, ferritin was lowered while 25 (OH)-vitamin D, albumin and alanine amino transferase (ALT) improved significantly postoperatively (Table 4). We found a high prevalence of patients with Parathyroid hormone (PTH) above reference-level both preoperatively and postoperatively. Hemoglobin-, folic acid-, cobalamine-, 1, 25-dihydroxyvitamin D (1,25(OH)_2_D), alkaline phosphatase (ALP), PTH and calcium levels did not change significantly after the procedure but 87% of the patients were taking vitamin and/or mineral supplements 24 months after the operation (Table 5). There was no difference in the vitamin or mineral status when comparing patients using supplements (n = 83) with patients not using supplements (n = 12) (chi-square test and t-test).

**Table 4:** Proportion of patients (%) with important biochemical variables below or above reference value prior to, and at 12 and 24 months after laparoscopic sleeve gastrectomy

**Variable**	**Preoperative**	**12 months**	**24 months**	**P value**		
				12 vs. 0 months	24 vs. 0 months	24 vs. 12 months
*No. with available data/total no:*	95-117/117	98-107/117	65-95/117			
	*No. below or above reference/no with available data (%)*					
Hb<ref. (11,5-16,0 g/dl)	7/117 (6%)	5/105 (4.8%)	4/95 (4.2%)	1	1	1
Ferritin<ref. (25-300 ug/l)	13/116 (11.2%)	20/101 (19.8%)	28/85 (32.9%)	0.021	0.001	0.118
Folic acid<ref. (> 5 nmol/l)	8/106 (7.5%)	8/100 (8.0%)	2/91 (2.2%)	1	0.453	0.125
Cobalamin<ref. (145–540 pmol/l)	4/111 (3.6%)	7/107 (6.5%)	3/94 (3.2%)	0.508	1	0.727
25(OH)D<ref. (30-150 nmol/l)	29/110 (26.4%)	5/105 (4.8%)	8/79 (10.1%)	<0.001	<0.001	0.289
25(OH)D<50 nmol/l)	73/110 (66.4%)	39/105 (37.1%)	24/79 (30.4%)	<0.001	<0.001	0.839
1,25(OH)_2_D>ref. (50-145 pmol/l)	0/104 (0.0%)	3/98 (3.1%)	1/65 (1.5%)	0.250	1	1
ALP>ref. (< 105 U/l)	6/116 (5.2%)	5/107 (4.7%)	7/94 (7.4%)	1	1	0.688
PTH>ref. (1.6-7.0 pmol/l)	35/95 (36.8%)	42/105 (40.0%)	36/85 (42.4%)	0.719	0.571	0.664
Calcium<ref. (2.15 – 2.55 mmol/l)	6/117 (5.1%)	1/108 (0.9%)	1/93 (1.1%)	0.124	0.125	1
Albumin<ref. (35-50 g/l)	16/117 (13.7%)	4/107 (3.7%)	8/94 (8.5%)	0.008	0.180	0.453
ALT>ref (35 U/l)	50/117 (42.7%)	11/107 (10.3%)	11/93 (11.8%)	<0.001	<0.001	1

No = Number. Hb = Hemoglobin. Ref = Reference value. 25(OH)D = 25 hydroxyvitamin D. 1,25(OH)_2_D = 1,25 dihydroxyvitamin D. ALP = Alkaline Phosphatase. PTH = Parathyroid hormone. ALT = Alanine aminotransferase.

**Table 5:** Number of patients (%) using supplements prior to, and at 12 and 24 months after laparoscopic sleeve gastrectomy

**Variable**	**Preoperative**	**12 months**	**24 months**
*no. with available data/total no:*	115/117	107/117	96/117
No supplement	99 (86.1%)	16 (15.0%)	12 (12.5%)
Multivitamin	11 (9.6%)	79 (73.8%)	70 (72.9%)
Cobalamin	4 (3.5%)	26 (24.3%)	29 (30.2%)
Folic acid	5 (4.4%)	23 (21.5%)	18 (18.8%)
Calcium	2 (1.8%)	28 (26.2%)	35 (36.5%)
Iron	2 (1.8%)	3 (2.8%)	16 (16.7%)

No = Number.

## Discussion

In the present study we find LSG to have acceptable morbidity-rates and to be an effective procedure for weight loss and resolution of comorbidities. LSG had high resolution rates for T2DM, hypertension, hyperlipidemia, sleep apnea, musculoskeletal pain, snoring, urinary leakage and amenorrhea. The BMI and the prevalence of obesity-related diseases were stable between 12 and 24 months postoperatively. Between 85 and 90% of patients were taking some kind of vitamin and/or mineral supplement at follow-up.

In general, the reported complication rates for LSG are low despite high surgical risks in this patient group [8]. Shi et. al. systematically reviewed major perioperative complications for LSG and found a mean ± SD of 1.17 ± 1.86% for leaks and 3.57 ± 5.15% for bleeding respectively [14]. In order to reduce our leak-rate we have become particularly careful not to use heat-creating instruments close to the stomach wall at the cardia where both leaks occurred. In an attempt to reduce bleeding, we have changed our regime for prophylaxis against thrombosis in that the prophylaxis is started postoperatively and at reduced dosage. Other measures that could influence the rate of bleeding would be the use of different stapler cartridges and buttress material. There is currently no clear consensus on how the surgical technique is optimally performed [14], which makes it even more important to continuously evaluate the results at different centers.

We are only presenting resolution and not changes in the degree of severity of the obesity-related diseases or conditions, these results therefore represent an underreporting of the patients’ improvement. Remission rates for T2DM, hypertension, hyperlipidemia and sleep apnea are higher among our patients than among sleeve- and gastric bypass operated patients in the study by Zhang et al., but our gastroesophageal reflux-rate is also higher [12]. This could be due to differences in the surgical technique as we used a somewhat smaller boogie (32 versus 38/40 Fr), and we start the resection closer to the pylorus. For infertility, we observed a reduction in infertility rate of 55.0% at two years, but as pregnancy was strongly discouraged for the first 12 months after the operation, the study is dependent on 36 months data for completion of the infertility data according to the definition. The regain of a normal menstrual cycle in all amenorrheic premenopausal females is remarkable.

Association between LSG and GERD has been systematically reviewed, finding both a significant increase and a significant decrease in GERD after LSG [15]. Our study shows a significant increase in GERD after the operation, even though five of our fifteen patients who were treated for GERD symptoms preoperatively had resolution of their GERD symptoms postoperatively. Our advice has been to have smaller meals at increased frequency and consume foods at slower rates with sufficient chewing, which might have some effect in reducing GERD-symptoms as Melissas et. al. have also experienced [16]. In accordance to the experience of Nocca et. al. [17] it is also our experience that the patients with GERD subjectively have a good effect of proton pump inhibitors. Howard et. al. [18], who had a one year GERD rate of 21.0%, declare that all of their GERD patients were “extremely happy with their surgery” and “would choose the procedure again”. Despite Howard et. al.´s findings, GERD is a potential drawback for the LSG and more work is being done in order to reduce the risk for GERD after LSG [15,19,20].

A high prevalence of micronutrient deficiencies among morbidly obese prior to bariatric surgery has been observed, a proposed consequence of malnutrition and/or altered bioavailability to micronutrients due to reduced dietary intake, reduced levels of hydrochloric acid and intrinsic factor [21]. The number of patients with ferritin levels below reference range in our data increased significantly, similar to the findings of Himpens et. al. [22]. Himpens et. al. also found cobalamin deficiency one and three years after LSG, which together with iron-related deficiencies are the most common deficiencies after bariatric surgery [22,23]. The number of patients with cobalamin-deficiency was not altered in our study, but 27% and 29% of the patients were substituted with folic acid or cobalamin respectively already one year after surgery. Unfortunately, we do not know whether this substitution was based on low serum values for these vitamins or not. Our findings highlight a need for further exploring the necessity of folic acid, cobalamin, iron and possibly calcium-substitution in LSG patients before making any general recommendations.

Values for albumin and ALT showed significant improvement after the operation, and ALT levels remained significantly lowered at 24 months. Obesity is associated with non-alcoholic fatty liver disease (NAFLD) [24], and resolution of NAFLD has been proven after bariatric surgery [25]. Improvement of liver-associated biochemical variables due to resolution of NAFLD is therefore a probable explanation of our finding.

LSG has been found to be equally as safe and effective for weight loss and resolution of comorbidities as the Roux-en-Y gastric bypass (RYGBP) in the short term [12,23], and as the small bowel is not transected and no mesenteric defects are created, the risk for long term complications as jejunal ulcers and internal hernias are avoided. Also, further conversion to BPDDS or RYGBP if inadequate weight loss or weight regain should occur makes LSG a good option among the bariatric procedures. Long term effects of LSG are, however, still limited in terms of possible weight regain, side effects and persistence of comorbidity resolution [14,17,26].

## Conclusion

We find LSG to have acceptable morbidity-rates and to be an effective procedure for weight loss and remission of obesity-related diseases. Further development of the technique should be attempted, particularly to reduce the risk for postoperative gastroesophageal reflux.

## Competing interests

Villy Våge has had travel expenses for two international conferences covered by Covidien and Johnson & Johnson. Vetle Aaberge Sande, Gunnar Mellgren, Camilla Laukeland, Jan Behme and John Roger Andersen do not have any commercial associations that might be a conflict of interest in relation to this article.

## Authors' contributions

VV was responsible for the surgery, collection, and extraction of data, and participated in the statistical analysis and writing of the article. VAS participated in the extraction and analysis of data, and in writing of the article. GM participated in the design of the study, analysis of the blood-samples and writing the manuscript. CL was responsible for the dietary advices to the patients and participated in writing the manuscript. JB operated about half of the patients and participated in writing the manuscript. JRA participated in the statistical analysis and writing of the article. All authors read and approved the final manuscript.

## Pre-publication history

The pre-publication history for this paper can be accessed here:

http://www.biomedcentral.com/1471-2482/14/8/prepub
